# High-intensity interval training improves cardiovascular and physical health in patients with rheumatoid arthritis: a multicentre randomised controlled trial

**DOI:** 10.1136/bjsports-2024-108369

**Published:** 2024-08-23

**Authors:** Annelie Bilberg, Kaisa Mannerkorpi, Mats Borjesson, Sara Svedlund, Jenny Sivertsson, Eva Klingberg, Jan Bjersing

**Affiliations:** 1 Institute of Neuroscience and Physiology, Department of Health and Rehabilitation, Physiotherapy, University of Gothenburg Sahlgrenska Academy, Goteborg, Sweden; 2 Department of Occupational and Physiotherapy, Sahlgrenska University Hospital, Goteborg, Sweden; 3 Institute of Medicine, Department of Molecular and Clinical Medicine, University of Gothenburg, Goteborg, Sweden; 4 Center for Lifestyle Intervention, Department of MGAÖ, Sahlgrenska University Hospital, Goteborg, Sweden; 5 Institute of Medicine, Department of Molecular and Clinical Medicine, University of Gothenburg Sahlgrenska Academy, Goteborg, Sweden; 6 Department of Clinical Physiology, Sahlgrenska University Hospital, Goteborg, Sweden; 7 Department of Physiotherapy, Uddevalla Hospital, Uddevalla, Sweden; 8 Institute of Medicine, Department of Rheumatology and Inflammation Research, University of Gothenburg Sahlgrenska Academy, Goteborg, Sweden

**Keywords:** Cardiovascular Diseases, Exercise, Physical fitness, Randomized Controlled Trial, Physical Therapy

## Abstract

**Objectives:**

Patients with rheumatoid arthritis (RA) have substantially elevated risk for cardiovascular diseases, and low cardiorespiratory fitness (VO_2_max) is a major mediator. The aim of this assessor-blinded, two-armed multicentre randomised controlled trial was to evaluate the effects of high-intensity interval training (HIIT) and strength exercise on cardiovascular health, physical fitness and overall health in patients with RA.

**Methods:**

In total, 87 patients (86% female; aged 20–60 years) were randomly assigned to an intervention group (IG) or a control group (CG). The IG performed HIIT and strength exercise for 12 weeks. The CG was instructed to be physically active on a moderately intensive level, ≥150 min/week. Primary outcome was change in VO_2_max. Secondary outcomes were changes in anthropometry measures, muscle strength, overall health (Visual Analogue Scale (VAS)-Global), Patient Global Impression of Change (PGIC), pain and disease activity (Disease Activity Score in 28 joints (DAS28)).

**Results:**

There was a significant mean group difference of change on VO_2_max (3.71 mL/kg/min; 95% CI 2.16, 5.25) in favour of the IG. Significant mean group differences of change were also seen for O_2_-pulse (1.38; 95% CI 0.85 to 1.91), waist circumference (−2.6; 95% CI −5.09 to –0.18), 1-minute sit-to-stand (5.0; 95% CI 3.35 to 6.72), handgrip strength (28.5; 95% CI 3.80 to 52.8), overall health (−14.7; 95% CI –23.8 to –5.50) and PGIC (p<0.0001) in favour of the IG. No significant mean group differences of change were found for pain (−4.0; 95% CI −13.07 to 5.06), DAS28 (−0.25; 95% CI −0.60 to 0.10) and erythrocyte sedimentation rate (−0.64; 95% CI −3.23 to 1.90).

**Conclusion:**

Supervised HIIT and strength exercise improved cardiovascular health, physical fitness and overall health without a deterioration in pain and disease activity and should be considered in patients with well-controlled RA.

**Trial registration number:**

NCT05768165.

**WHAT IS ALREADY KNOWN ON THIS TOPIC**

- Patients with rheumatoid arthritis (RA) have an elevated risk for cardiovascular diseases compared with the general population.
- Exercise has the potential to improve cardiovascular health in RA, although only a minority of patients engage in exercise on a high enough level to improve cardiorespiratory fitness.

**WHAT THIS STUDY ADDS**

- Supervised high-intensity interval training (HIIT) and strength exercise for 12 weeks significantly improved VO_2_max, waist circumference, muscle strength and overall health without accentuating disease activity and pain in patients with well-controlled RA.

**HOW THIS STUDY MIGHT AFFECT RESEARCH, PRACTICE OR POLICY**

- Supervised HIIT and strength exercise appear to be feasible and well tolerated by patients and could be recommended to improve cardiovascular and physical health in patients with well-controlled RA.

## Introduction

 Rheumatoid arthritis (RA) is a common chronic autoimmune disease characterised by systemic inflammation and peripheral arthritis.^1 2^ Clinical symptoms, such as pain, stiffness and fatigue, often lead to functional disability^3^ and deterioration in health.^4^ Patients with RA have an increased prevalence of comorbidities,^5^ mainly cardiovascular diseases (CVDs).^6^ The elevated CVD risk of RA is comparable to that of type 2 diabetes mellitus.^7^ Increased cardiovascular morbidity and mortality in RA have partially been attributed to myocardial infarction and congestive heart failure, caused by atherosclerotic events.^6^

The CVD risk is linked to the systemic inflammatory process in combination with increased levels of traditional risk factors.^8 9^ Physical inactivity has been associated with an adverse CVD risk profile,^10^ and cardiorespiratory fitness (CRF) is similarly inversely associated with CVD morbidity and mortality in RA.^11^ Exercise shows beneficial effects on CRF,^12^ disease activity,^13^ possibly diminishes other cardiovascular risk factors in RA^14^ and is recommended as part of patient standard care.^15^ However, time restriction, pain and fatigue are common barriers for exercise in individuals with RA,^16^ and only a minority of patients engage in exercise at high enough intensity to improve CRF.^10^

A time-efficient method to improve cardiorespiratory fitness is high-intensity interval training (HIIT), which involves alternating bouts of high intensity on 90–95% heart rate maximum (HRmax) and periods of lower intensity.^17^ The exercise mode is suggested to result in greater improvements in VO_2_max than moderately continuous exercise.^18^ This is important when aiming to prevent CVD since the risk for CV morbidity and mortality decreases for every increment in 1 mL of VO_2_max.^19^ However, to date, the evidence of HIIT on cardiovascular health in RA is scarce.^20^ Cardiorespiratory exercise in combination with muscle strength exercise has been recognised to have the largest potential to reduce CV mortality^21^ and to improve health.^22^ Hence, the primary aim of this study was to evaluate the effects of HIIT and strength exercise on cardiorespiratory fitness. The secondary aim included changes in muscle strength, anthropometry measures, lipid status, overall health, Patient Global Impression of Change (PGIC), pain and disease activity (Disease Activity Score in 28 joints (DAS28)).

## Methods

### Design

The study was designed as an assessor-blinded, two-armed multicentre randomised controlled trial comparing the effects of 12-week supervised high-intensity exercise with health-promoting physical activity.

### Participants

Patients with RA were recruited from the rheumatology departments at Sahlgrenska University Hospital and Uddevalla Hospital through the Swedish Rheumatology Quality Register. The recruitment, intervention and data collection were performed between August 2021 and May 2023. Patients fulfilling the American College of Rheumatology/European Alliance of Associations for Rheumatology (ACR/EULAR) 1987/2010 criteria for RA,^23 24^ with disease duration >1 year, aged 20–60 years, stable treatment on antirheumatic drugs for the past 3 months, and low-to-moderate disease activity (DAS28 <5.1) were eligible for inclusion. DAS28 <5.1 was chosen as upper limit for inclusion for safety considerations. Exclusion criteria were symptoms of CVD, other comorbidities, physical disabilities and pregnancy precluding participation in high-intensity exercise, engagement in regular cardiorespiratory exercise on high intensity level (>1 hour/week for the past 6 months), inability to understand and speak Swedish.

The study complied with the Declaration of Helsinki. Informed written consent was obtained from all patients before baseline examinations. The trial was registered prospectively on ‘FoU in Sweden’ (Research and Development in Sweden) (registration number: 275642) and retrospectively on ClinicalTrials.gov (NCT 05768165) (online supplemental file 1).

### Adverse effects and safety

An initial prescreening was performed to exclude patients with chronic coronary syndrome and severe comorbidities by following a protocol modified from the European Society of Cardiology’s (ESC) guidelines.^25^ At the screening visit at the Sahlgrenska University Hospital, the patient’s medical health status was assessed, and disease activity was evaluated by a rheumatologist, followed by a cardiopulmonary exercise test (CPET) further screening of contradictions to participate in high-intensity exercise.^25^ Adverse effects during exercise were defined as severe symptoms related to the cardiopulmonary system, that is, exercise-induced chest pain, palpitation/arrhythmias, dyspnoea, nausea, dizziness or severe discomfort. Also, increased pain which could be related to exercise or increased disease activity was reported.

### Exercise intervention

The exercise protocol is described in detail in table 1. Patients allocated to the intervention group (IG) participated in 12-week exercise intervention, following the American College of Sports Medicine (ACSM) recommendations for cardiorespiratory and muscle strength exercise.^26^ The supervised exercise was conducted in a hospital setting, in groups of eight. Two sessions per week were supervised face-to-face by a physiotherapist, and one aerobic session (70% HRmax) of the patient’s own choice per week was non-supervised. The heart rate (% HRmax) during all cardiorespiratory exercises was monitored continuously with an individual heart rate sensor (Polar H10) strapped around the chest and connected to an exercise application (Polar Beat) on the patient’s cellphone.

**Table 1:** Description of the exercise protocol

Exercise protocol	
The exercise programme followed the American College of Sports Medicine exercise recommendations. Individually tailored according to physical capacity and physical ability. Exercise guidance followed the principles of self-efficacy and motivational support, applying supervision, identification of possible limitations and barriers, an individual exercise diary, and continuous dialogue with individual feedback by the physiotherapist.	
High-intensity interval training	
Delivery	Supervised by a physiotherapist at the physiotherapeutic facility at a hospital
Type	Exercise on a cycle ergometer
Frequency	Two times per week
Intensity	10 min warm up at 60–70% of HRmax. 4×4 min interval exercise at 90–95% HRmax with 3 min of active recovery period at 70% HRmax between each interval. 3 min cool down at 60–70% HRmax. The intensity was controlled by a heart rate sensor (Polar H10) connected to an exercise app on the patient's cellphone during each session. The individual HRmax was determined at the end of the CPET at baseline.
Progression	The intensity of the cardiorespiratory exercise was gradually increased to the pulse target zone (90–95% HRmax) over the first 2 weeks, depending on the subject’s adaptation to the exercise protocol.
Time	38 min
Muscle strength exercise	
Delivery	Supervised by a physiotherapist at the physiotherapeutic facility at a hospital
Type	Exercises of large muscle groups. A total of eight exercises, individually adapted. Examples of exercises: leg press, squat, hip extension, pull down, biceps curl with free weights, rows to chest, sit ups, lumbar back extension
Frequency	Two times per week
Repetition	8–10
Sets	2–3
Progression	Weeks 1–2: 50–60% of 1RM, 15 repetition, 1–2 setsWeeks 3–6: 60–70% of 1RM, 8–10 repetition, 2–3 setsWeeks 7–12: 70–80% of 1RM, 8–10 repetition, 2–3 sets
Time	20 min
Non-supervised aerobic exercise	
Delivery	Individual exercise of the patient’s own choice
Type	Walking/running/bicycling, outdoor or at fitness centre
Frequency	One day per week
Intensity	70% of HRmax, including 10 min of warm-up on lower intensity, controlled by a heart rate sensor (Polar H10) connected to an exercise app on the patient's cellphone or pulse watch.
Time	40 min
**Control group**Individual information of the general recommendations for physical activity, with encouragement to be physically active on moderate intensity ≥150 min/week.**Home exercise**A total of five exercises including muscle strengthening of lower extremities and trunk and one leg standing, without any exercise equipment.	

CPETcardiopulmonary exercise testHRmaxheart rate maximum1RMone repetition maximum

The HIIT sessions on cycle ergometers included high-intensity bouts (90–95% HRmax) alternated with periods on lower intensity (70% HRmax), repeated four times. The intensity of the cardiorespiratory exercise was gradually increased to the pulse target zone over the first 2 weeks, depending on the participant’s adaptation to the HIIT protocol.

This was directly followed by strength exercise involving large muscle groups, for 20 min: eight exercises were performed with free weights, weight machines and body weight. The dose was 2–3 sets, 8–10 repetitions, at 70–80% of one repetition maximum (1RM). A progression of the workload was performed following a standardised protocol.

Th exercise was individually tailored based on the patient’s physical capacity and physical ability and modified according to present symptoms and health at the time of each session. Rated pain ≤5 on a Visual Analogue Scale (VAS) (0–10) was considered acceptable during exercise. Temporary workload modification was made if pain was >5 or persisted for more than 24 hours. In case of a rheumatological ‘flare-up’ or exacerbation, (eg, joint effusions), the patient was referred to the rheumatology department for consultation.

Adherence to the exercise protocol and the non-supervised sessions, duration and HRmax was recorded in the patient’s exercise diary. The details were discussed with the physiotherapist at the following supervised session. Attendance at the supervised sessions was registered by the physiotherapist.

Four physiotherapists with an expertise in rheumatology and trained in cardiopulmonary resuscitation supervised the exercise. Before startup, the physiotherapists attended a 6-hour education session on the exercise intervention, motivational support^27^ and self-efficacy approach^28^ and were given a treatment manual to follow.

### Control group

Patients allocated to the control group (CG) received individual information of the general health recommendations for physical activity, with encouragement to be physically active on moderate intensity level ≥150 min/week. A home exercise programme was provided, and verbal and written instructions were given (table 1).

All patients received standard outpatient care during the study period at their respective hospitals.

### Outcomes

Outcomes were assessed at baseline and immediately after the intervention at 3 months and included questionnaires addressing demographics, comorbidities, medication use and overall health, along with a medical examination, performance-based tests and blood samples. All assessors including physicians, physiotherapists, nurses and testing personnel for the CPET were blinded to group allocation.

#### Primary outcome and end criteria

Changes in cardiorespiratory fitness, VO_2_max (mL/kg/min), were selected as primary outcome. Cardiorespiratory fitness was assessed with a CPET on a bicycle ergometer, the standard procedure in Sweden. Weight-adjusted VO_2_max (mL/kg/min) was obtained during progressively increasing workload, during which gas exchange was analysed, following a protocol modified from the American Heart Association (AHA) guidelines.^29^ Simultaneously, recording of 12-lead ECG data, heart rate, blood pressure and rating scores of symptoms (BORG CR-10 scale) was assessed. A respiratory exchange ratio (VCO_2_/VO_2_) ≥1.10 in combination with a plateau in VO_2_ despite increased workload was used as the criterion for reaching VO_2_max. VO_2_max was calculated as the mean of the three highest consecutive 10-second measurements. The maximal obtained heart rate during the test was used as a reference during the cardiorespiratory exercises.

#### Secondary outcomes

VO_2_ (mL/min) and ventilatory maximum (VEmax, L/min) were assessed, oxygen pulse (O_2_-pulse) representing the product of ventricular stroke volume was calculated using the following formula: VO_2_ mL/min divided by HRmax.^30^ Resting blood pressure was recorded with an ambulatory blood pressure monitor with the patient in a seated position, where the lowest value out of two was recorded.

The 1-minute sit-to-stand test was used to assess muscle strength and endurance of the lower extremities.^31^ The number of completed rises from a standard chair in 60 s was recorded. Isometric handgrip strength was measured with a digital electronic dynamometer, the Grippit (AB detector, Gothenburg, Sweden), which measures grip strength in newtons (N).^32^ The average mean grip strength was used for assessment. Handgrip strength has been found to be associated with shoulder-arm strength in RA^33^; thus, handgrip strength was used as a surrogate measure to reflect the strength of the upper extremity.

Anthropometry was assessed with waist circumference, body weight and body height. Body mass index (BMI) (kg/m^2^) was calculated.

Blood samples were drawn after 8 hours of fasting. Lipid status was assessed by serum levels of triglycerides, high-density lipoprotein (HDL), low-density lipoprotein (LDL) and total cholesterol (TC). Inflammatory markers were assessed by high sensitivity C-reactive protein (CRP) and erythrocyte sedimentation rate (ESR).

Disease activity was estimated with the Disease Activity Score (DAS28), based on clinical assessment of 28 joints (swollen and tender), patient’s rating of health (VAS) and ESR.^34^ A DAS28 score≥2.6<5.1 indicates low-to-moderate disease activity and <2.6 indicates remission.

Physical activity at baseline was assessed with the Leisure Time Physical Activity Instrument, which assesses the amount of physical activity during the last week.^35^

Pain and overall health related to the rheumatological disease during the past weeks were rated on a VAS (0–100 mm), VAS-pain and VAS-global, where a higher score indicates worse pain or health.

Changes in symptoms were assessed with the Patient Global Impression of Change (PGIC) questionnaires.^36^

### Patient involvement

A patient research partner from the Swedish Rheumatism Association was involved in the study design, the prescreening questionnaire and choice of outcome measures.

### Equity, diversity and inclusion

Our clinical trial includes women and men with RA of different ages and demographics. All eligible patients were considered for participation. More women than men volunteered to participate. While no diversity in study population was planned to balance for gender, ethnicity, socioeconomic level or representation from marginalised groups, the recruitment area encompassed a broad range of socioeconomic groups. We acknowledge that our study excluded patients with such physical disabilities and comorbidities precluding participation in high-intensity exercise. Our research and author team includes both women and men from different professional disciplines and levels.

### Randomisation

Randomisation was performed after screening and enrolment, with optimal allocation (minimisation) using a computerised algorithm in order to balance for sex, age, VO_2_max (mL/kg/min) and study site. Patients were informed of their group allocation by the physiotherapist supervising the exercise.

### Statistical analyses

Descriptive statistics are presented as mean and standard deviation (SD), frequency and percentages. Comparisons between groups were performed with the Fisher’s non-parametric permutation test for continuous variables and Manthel-Haenszel χ^2^ test for categorical variables. The main statistical analysis was the analysis of covariance (ANCOVA) of the change for the primary outcome, adjusted for baseline variables for age, VO_2_max and sex. Primary efficacy analyses were performed on intention-to-treat (ITT) population using multiple imputation for missing values. The effect size was calculated as Cohen’s d coefficient. A per protocol analysis on patients in the IG that followed ≥70% of the supervised exercise sessions and the patients in the CG was conducted for primary and secondary outcomes. An exploratory interaction analysis was conducted with the primary effect variable as dependent and the interaction between randomised groups and selected baseline variables. Additionally, an unadjusted analysis was conducted for comparisons between groups based on sex for key outcomes. All tests were two-tailed and conducted at 0.05 significance level. All analyses were performed using SAS V.9.2 (Cary, North Carolina, USA).

### Sample size

To achieve 80% power to detect 10% difference of change in weight-adjusted VO_2_max with a baseline value of 34.5 mL^37^ and estimated SD difference of 5.0, at 5% significance level, a total of 70 patients were needed. To compensate for a dropout rate of 20%, 88 patients were aimed to be recruited.

## Results

**Figure 1:**
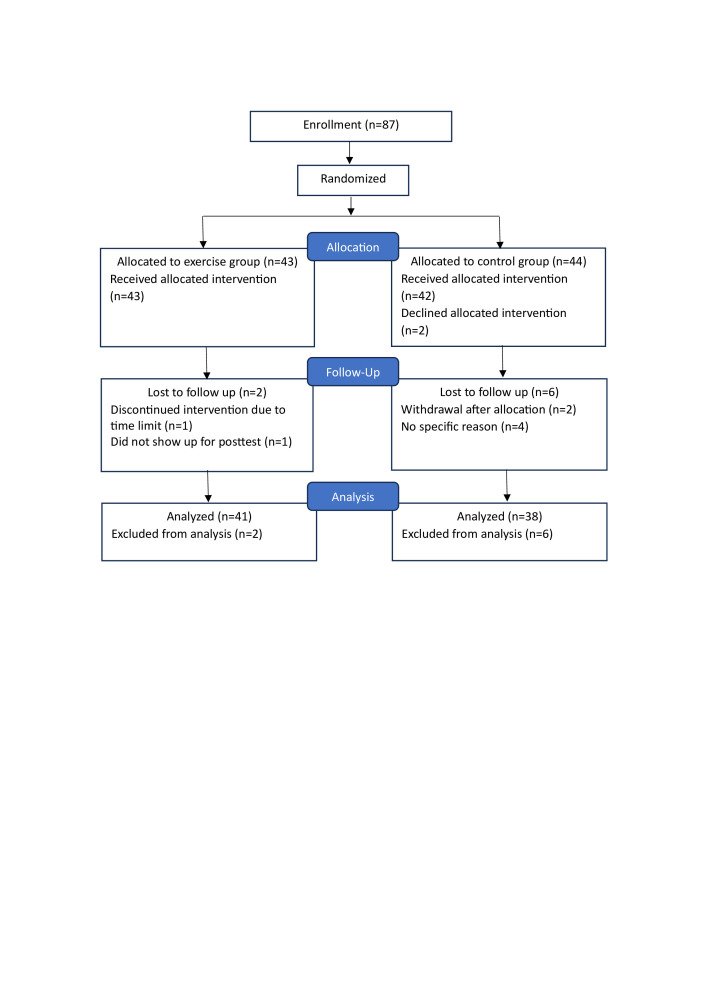
Consolidated Standards of Reporting Trials (CONSORT) diagram for the two groups in the randomised control trial.

In total, 87 patients were included in the study (figure 1), of whom 23% had a low-to-moderate disease activity and 77% were in remission, at baseline. The disease activity did not change during the study. The groups were considered similar in demographic and clinical characteristics at baseline (table 2).

**Table 2:** Baseline demographic and clinical characteristics for the intervention group and the control group with rheumatoid arthritis

	Intervention group(n=43)	Control group(n=44)	P value
Sex, women/men	37 (86.0%)/ 6 (14.0%)	36 (81.8%)/8 (18.2%)	0.59
Age, years	48.4 (10.1)	47.9 (9.3)	0.83
Disease duration, years	6.1 (4.58)	6.9 (5.37)	0.44
Civil status			0.59
Married, cohabitant	29 (67.4%)	32 (72.7%)	
Employment status			0.57
Working	41 (95.3%)	42 (95.4%)	
Studies	1 (2.2%)		
Education, years			0.94
≤12 years	14 (32.6%)	13 (29.5%)	
Postgraduate high school	4 (9.3%)	4 (9.1%)	
College, University	25 (58.1%)	27 (61.4%)	
Tobacco			0.64
Current smoker	6 (14.3%)	8 (18.6%)	
Former smoker	10 (23.8%)	7 (16.3%)	
MVPA, hours/week	2.1 (1.58)	3.1 (2.97)	0.34
VO_2_max, mL/kg/min	26.2 (5.3)	26.4 (6.5)	0.87
Grip strength	214.6 (78.1)	213.1 (87.4)	0.92
1-minute STS	23.7 (5.5)	25.0 (6.4)	0.31
Anthropometry			
BMI, kg/m^2^	27.1 (5.3)	27.1 (5.3)	1.0
Length, cm	171.5 (7.8)	169.8 (9.7)	0.36
Weight, kg	79.4 (15.2)	78.5 (19.1)	0.80
Waist circumference	89.7 (13.6)	89.1 (15.2)	0.83
Disease activity			
DAS28	2.0 (0.90)	2.0 (1.18)	0.83
Tender joints	0.6 (1.91)	1.0 (4.30)	0.29
Swollen joints	0.8 (1.59)	0.5 (1.00)	0.29
ESR, mm/hour	11.0 (11.2)	11.7 (10.1)	0.75
CRP, mg/L	2.2 (3.00)	2.3 (3.07)	0.90
VAS-global	21.1 (18.2)	18.5 (19.0)	0.51
VAS-pain	20.2 (17.6)	20.1 (20.1)	0.97
Medication			
Conventional DMARD	34 (79.1%)	32 (72.7%)	0.49
Biological DMARD	23 (53.5%)	21 (47.7%)	0.59
Targeted synthetic DMARD	4 (9.3%)	3 (6.8%)	0.67
Corticosteroids (oral)	1 (2.3%)	2 (4.5%)	0.57
NSAID	17 (39.5%)	18 (40.9%)	0.90
Anti-hypertensives	4 (9.3%)	4 (9.1%)	0.97
Statins	1 (2.3%)	1 (2.3%)	0.99

Values are presented as mean (SD) and number (%). MVPA, leisure time physical activity on moderate-to-vigorous intensity;

BMIbody mass indexCRPC-reactive proteinDAS28Disease Activity Score in 28 jointsDMARDdisease-modifying antirheumatic drugESRerythrocyte sedimentation rate1-minute S-T-S1-minute sit-to-stand testMVPAleisure time physical activity on moderate-to-vigorous intensityNSAIDnon-steroidal anti-inflammatory drugVASVisual Analogue ScaleVO_2_maxmaximal weight corrected oxygen uptake

### Adherence

The patients in the IG attended an average of 19 (SD 6.0) supervised sessions and performed an average of 12 (SD 3.6) self-administered sessions. A total of 33 patients in the IG followed ≥70% of the supervised exercise sessions. The dropout rate during the intervention period was eight in total, seven women and one man, all significantly younger than the rest of the study population. In the IG, one patient discontinued the study after attending the first supervised session and one patient did not show up for post-test at month 3. In the CG, two withdrew when receiving group allocation and four did not show up for post-test (figure 1).

### Adverse effect and safety

In the IG, one patient experienced irregular heart rate during a supervised HIIT session. After consultation with the cardiologist and referral to UCG for evaluation of arrythmia, the patient could complete the intervention at moderately intensive level. Four patients encountered temporarily increased musculoskeletal pain during the strength exercise, which was managed with temporary exercise modification. One patient experienced persistent musculoskeletal pain after half of the intervention period and completed the intervention without the strength exercise. Two patients received a corticosteroid injection each, one in the ankle and one in the wrist, followed by temporary exercise modification.

### Primary outcome

A significant mean group difference of change was found for VO_2_max (3.71 mL/kg/min, 95% CI 2.16 to 5.25, p<0.0001) where the IG showed significant improvements compared with the CG, adjusted for sex, age and baseline VO_2_max. The sensitivity analysis on the ITT population revealed an equal mean difference of change for VO_2_max between the groups (table 3).

**Table 3:** Changes in primary outcome from baseline (BL) to 3 months (3M) follow-up between the groups for the patients with rheumatoid arthritis

	Intervention group(n=43)		Control group(n=44)		Between group	Effect size
	BLMeans (SD)	3MMeans (SD)	BLMeans (SD)	3MMeans (SD)	LS means difference of changeBL to 3M (95% CI)P value	
VO_2_max, mL/kg/min*	26.2 (5.30)	29.2 (6.70)	26.4 (6.50)	25.7 (5.90)	3.71 (2.16 to 5.25)<0.0001	1.06
ITT						
VO_2_max, mL/kg/min†					3.71 (2.16 to 5.25)<0.0001	
Per protocol						
VO_2_max, mL/kg/min‡	26.8 (5.30)	30.1 (6.60)	26.4 (6.50)	25.7 (5.90)	4.1 (2.43 to 5.77)<0.0001	1.14

Values are shown as mean and SD, unless indicatinged otherwise. VO2max; weight corrected maximal oxygen uptake.

* Primary analysis adjusted for BL age, VO_2_max and sex.

† Multiple stochastic imputation of 100 dataset adjusted for the minimizsation variables.

‡ Complementary analysis including patients attending ≥70% of supervised exercise sessions (n=33), adjusted for BL age, VO_2_max and sex.

VO_2_maxweight-corrected maximal oxygen uptake

### Secondary outcomes

**Figure 2:**
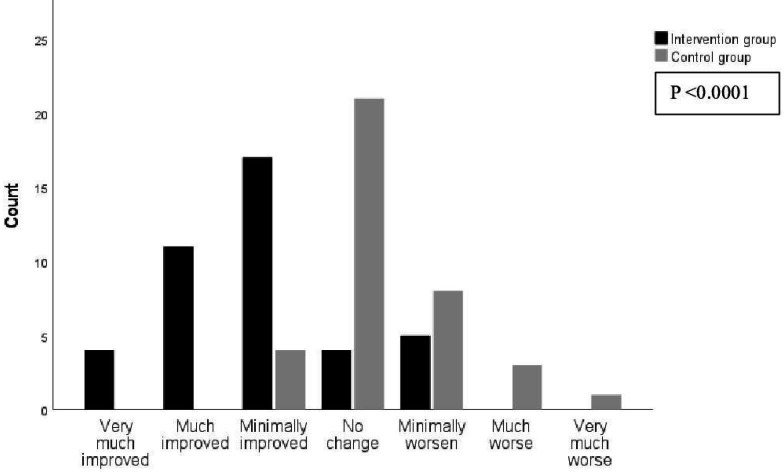
Rating of Patient’s Global Impression of Change after 12 weeks for the intervention group and the control group.

A significant mean group difference of change was found for VO_2_ mL/min and O_2_-pulse (p<0.0001) in favour of the IG (table 4). A significant mean group difference of change was also found for handgrip strength (p=0.021) and leg muscle strength (p<0.001) where the IG had a better muscle function compared with the controls. In addition, a significant mean group difference of change was found for waist circumference (p=0.035) and overall health (p=0.001) in favour of the IG (table 4). The PGIC rating was significantly different (p<0.0001) between the groups at month 3, with very much to minimally improved symptoms among 78% in the IG and 11% in the CG (figure 2).

**Table 4:** Changes in secondary outcomes from baseline to 3 months follow-up between the groups for the patients with rheumatoid arthritis

	Intervention group(n=43)		Control group(n=44)		Between group	Effect size
	BLMeans (SD)	3 monthsMeans (SD)	BLMeans (SD)	3 monthsMeans (SD)	Mean difference of change BL to 3 months (95% CI)P value	
VO_2_, mL/min	2047.0 (435.0)	2282.0 (576.0)	2013.0 (569.0)	1988.0 (579.0)	260.6 (168.5 to 351.5)<0.001	1.31
O_2_-pulse, mL/beat/min	11.8 (2.5)	13.3 (3.4)	11.9 (3.1)	12.1 (3.4)	1.38 (0.85 to 1.91)<0.001	1.19
VEmax, L/min	86.1 (18.4)	91.1 (21.3)	82.1 (22.2)	81.2 (24.9)	6.73 (1.21 to 12.20)0.016	0.57
RER	1.2 (0.09)	1.17 (0.09)	1.2 (0.09)	1.2 (0.10)	−0.01 (−0.04 to 0.02)0.45	0.17
HRmax, beats/min	173.4 (11.5)	172.1 (11.8)	169.4 (16.5)	164.8 (16.3)	1.56 (−1.79 to 4.84)0.37	0.57
Systolic BP	123.0 (17.8)	120.7 (16.6)	123.9 (15.9)	124.4 (17.7)	−1.98 (−7.74 to 3.79)0.49	0.16
Diastolic BP	74.8 (12.1)	73.3 (10.2)	74.5 (10.5)	75.4 (11.3)	−1.72 (−5.00 to 1.56)0.31	0.23
Grip strength, N	214.6 (78.1)	249.8 (89.6)	213.1 (87.4)	216.8 (93.0)	28.5 (3.80 to 52.80)0.021	0.52
1-minute STS, no	23.7 (5.5)	30.4 (5.3)	25.0 (6.4)	25.9 (6.7)	5.0 (3.35 to 6.72)<0.001	1.33
Anthropometry measure						
Weight, kg	79.4 (15.2)	78.9 (14.6)	78.5 (19.1)	78.2 (19.2)	−0.44 (−1.32 to 0.44)0.33	0.23
BMI, kg/m^2^	27.1 (5.3)	27.0 (5.1)	27.1 (5.3)	26.9 (5.1)	−0.13 (−0.43 to 0.17)0.39	0.20
Waist circumference, cm	89.7 (13.6)	86.8 (11.6)	89.1 (15.2)	88.7 (14.4)	−2.60 (−5.09 to −0.18)0.035	0.47
Serum lipids						
S-TC, mm/L	5.30 (1.32)	5.20 (1.31)	5.16 (1.00)	5.22 (0.95)	−0.14 (−0.35 to 0.08)0.21	0.29
S-HDL, mm/L	1.61 (0.40)	1.61 (0.37)	1.58 (0.39)	1.59 (0.39)	−0.02 (−0.10 to 0.07)0.72	0.08
S-LDL, mm/L	3.58 (1.19)	3.45 (1.20)	3.45 (0.89)	3.47 (0.78)	−0.08 (−0.27 to 0.10)0.39	0.20
S-TG, mm/L	0.92 (0.42)	0.95 (0.42)	0.99 (0.42)	0.95 (0.38)	0.01 (−0.11 to 0.13)0.88	0.04
Disease activity						
DAS28-ESR	2.0 (0.90)	2.0 (0.82)	2.0 (1.18)	2.3 (1.33)	−0.25 (−0.60 to 0.10)0.16	0.32
ESR	11.0 (11.2)	12.2 (10.4)	11.7 (10.1)	13.5 (11.4)	−0.64 (−3.23 to 1.90)0.64	0.11
CRP	2.2 (3.00)	2.4 (3.49)	2.3 (3.07)	2.82 (3.33)	−0.21 (−1.56 to 1.16)0.77	0.07
VAS-global, 0–100	21.1 (18.2)	18.3 (16.8)	18.5 (19.0)	29.5 (26.9)	−14.7 (−23.8 to −5.5)0.001	0.72
VAS-pain, 0–100	20.2 (17.6)	19.5 (17.1)	20.1 (20.1)	21.6 (22.4)	−4.0 (−13.07 to 5.06)0.38	0.20

Values are shown as mean and SD, unless indicatinged otherwise.

Missing values at month 3 in total for the IG (n=2); Missing values at month 3 in the IG; CRF (), BP (), grip strength (), STS (), Serum lipids (), body measures (), DAS28 (), ESR and CRP (), VAS-global and VAS-pain ()Mmissing values at month 3 in the CG; CRF (n=8), BP (n=6), grip strength (n=6), STS (n=6), Sserum lipids (n=6), body measures (n=6), DAS28 (n=7), ESR and CRP (n=6), VAS-global and VAS-pain (n=7).

BMIbody mass indexBPblood pressure at restCRPC-reactive proteinDAS28Disease Activity Score in 28 jointsESRerythrocyte sedimentation rateHRmaxmaximal heart rate1-minute STS1-minute sit-to-stand testO_2_-pulseoxygen pulseRERrespiratory exchange ratioSerum levels of S-TGtriglyceridesS-HDLhigh-density lipoproteinS-LDLlow-density lipoproteinS-TCtotal cholesterolVEmaxventilatory maximalVO_2_, mL/minmaximal oxygen uptake

The exploratory interaction analysis is presented in online supplemental table 4.

### Per protocol analysis

The per protocol analysis for the primary outcome variable adjusted for sex, age and baseline VO_2_max revealed a significant mean group difference of change for VO_2_max (4.1 mL/kg/min; 95% CI 2.43 to 5.77), p<0.0001 in favour of the patients that followed ≥70% of the supervised sessions (table 3). For the per protocol analyses of the secondary outcomes, see online supplemental table 5. The subgroup analysis of key outcome variables based on sex is presented in online supplemental table 6.

## Discussion

The 12-week intervention of HIIT and strength exercise showed beneficial treatment effects on patients’ cardiovascular health in terms of significant improvements in VO_2_max, O_2_-pulse and reduction in abdominal fat. Moreover, significant treatment effects were also found on muscle strength and overall health, showing additional beneficial health effects for the patients in the IG. Importantly, the disease activity and pain did not deteriorate, implying a tolerance to the exercise protocol.

The mean treatment effect of 3.71 mL in VO_2_max indicates substantial health-related gains for the patients in the IG since the risk of all-cause mortality and CVD mortality has been found to be decreased by 12–13%^38 39^ and 13%, respectively,^38^ per increase in 3.5 mL/kg/min of VO_2_max. The treatment effect in VO_2_max is comparable with the findings from two previous studies of axial spondyloarthritis^40^ and psoriatic arthritis^41^ (2.7–3.7 mL/kg/min), with similar cardiorespiratory exercise intensity and volume. Compared with our findings, a study conducted in patients with RA reported a smaller treatment effect on VO_2_max after 20 weeks of cardiorespiratory and strength exercise, three times per week.^42^ The differences could partly be attributed to the intensity of the cardiorespiratory exercise since this was lower compared with the one in our study. In the present study, the observed increase in VO_2_max average in the IG correspond to a 11% improvement (12.6% difference in change between the groups), implying a clinically relevant effect. Additionally, the improvement in VO_2_max was accompanied by increase in O_2_-pulse which has been found to reflect increased left ventricular stroke volume,^43^ a key factor when VO_2_max is improved from cardiorespiratory exercise on a high intensity.^44^

The mean treatment effect on waist circumference indicates a reduction of abdominal fat, thereby loss of visceral fat mass in the IG. Although an indirect measure, a reduction of >2 cm in waist circumference is found to improve metabolic health profile even in the absence of weight los*s*.^45^ Exercise has the potential to reduce visceral adiposity in a dose-dependent manner, and both cardiorespiratory and strength exercise have shown to reduce body fat mass.^46 47^ The improvement by 16% in upper body strength and 30% in lower body strength in the IG point to a reduction in body fat mass and gain in muscle mass, align with the findings of a study with progressive strength exercise in RA.^48^

No major effects were found in serum lipids after 3 months. One explanation could be that the lipids were already within recommended levels at baseline and therefore the potential for improvement in these measures was limited. Our findings are in agreement with a recently published study of HIIT with well-controlled patients with inflammatory joint diseases.^49^ Still, HIIT has been found to improve the lipid profile in other non-rheumatic populations.^50 51^ Moreover, a non-randomised study of patients with RA reported improvement in blood lipids after 6 months with cardiorespiratory and resistance exercise, indicating that the duration of the intervention is also critical for possible changes in lipids.^52^ Thus, the potential effects of HIIT on lipid status in rheumatic diseases warrant more research with interventions of longer duration.

Overall health remained stable in the IG while it decreased in the CG. These findings together with a significant improvement in patients’ impression of change for the IG support the positive findings of the exercise intervention on the patients’ overall well-being and health.

No adverse effects were reported except a temporary increase in musculoskeletal pain for a few patients in the IG. Moreover, the adherence to the protocol must be considered as satisfactory where almost 80% of the patients followed ≥70% of the supervised sessions. Importantly, the treatment effect on VO_2_max (4.1 mL/kg/min) was even larger for the patients that followed the protocol. Our results support the evidence of HIIT as a superior method to maximise VO_2_max^18^ and add to the evidence for structured exercise on CVD managements in RA.^8^

### Strength and limitations

A major strength of this study is the randomised controlled design, blinding of all assessors for group allocation, objective measure of cardiorespiratory fitness and the exercise protocol, individually tailored based on the patient’s physical capacity and ability. Also, a strength is that the IG was compared with an active CG who received counselling on the general recommendations for physical activity regarding moderate intensity, a level recommended as the minimum to obtain health benefits. However, self-administered physical activity was not reported in the CG which is a limitation. The 1-minute sit-to-stand test does not predominantly assess muscle strength which could be considered a limitation. However, the test was chosen as it is easily applicable in a clinical setting with no requirement of advanced laboratory equipment. The majority of the study patients were in remission or had a low disease activity which is consistent with modern pharmacological treatment in RA, indicating a representative study sample.^53^ Due to few included men in the study, the results should be interpreted with some caution with regard to men. Nevertheless, the exploratory interaction analysis revealed that men had the highest potential to improve VO_2_max in the study.

## Conclusions

The supervised exercise intervention for 12 weeks displayed a number of beneficial and clinically significant effects on patients’ cardiovascular health, physical fitness and overall health without deterioration in disease activity and pain. HIIT and strength exercise in combination appear to be feasible and well tolerated and could be recommended as a treatment option to improve cardiovascular and physical health in patients with well-controlled RA.

## supplementary material

10.1136/bjsports-2024-108369 online supplemental file 1

10.1136/bjsports-2024-108369 online supplemental file 2

10.1136/bjsports-2024-108369 online supplemental file 3

10.1136/bjsports-2024-108369 online supplemental file 4

## Data Availability

Data are available upon reasonable request.
