# Supplementary material for: High-intensity interval training improves cardiovascular and physical health in patients with rheumatoid arthritis: a multicentre randomised controlled trial
Source: Br J Sports Med. 2024 Aug 23;58(23):e108369. doi: 10.1136/bjsports-2024-108369 (PMC11672065; doi:10.1136/bjsports-2024-108369)
Supplement: online supplemental file 1 [file bjsports-58-23-s001.pdf]

Expected benefit of high-intensity interval aerobic exercise program of 3 months for patients with RA: Improve cardiopulmonary function and health. Prevent deterioration in cardiovascular health and quality of life.

Rheumatoid arthritis (RA) is a chronic inflammatory disease with 0.7% prevalence rate in Sweden. RA is characterized by symmetrical inflammation of peripheral joints, although larger joints can also be affected. Clinical symptoms of RA are pain, stiffness, fatigue, limited range of motion in joints, and reduced muscle strength. Patients with RA have increased prevalence rates of comorbidities, mainly cardiovascular diseases and atherosclerosis. Despite more effective control of inflammation by improved pharmacological therapies, reduced physical function, cardiovascular disease and increased arterial stiffness remain major problems in RA.

Cardiovascular disease. Patients with RA have markedly increased risk for cardiovascular disease, leading to increased mortality[1]. Increased mortality in RA has partially been attributed to cardiac insufficiency and myocardial infarction[1, 2]. Cardiopulmonary and respiratory functions are assessed by means of cardiopulmonary exercise test (CPET). A low maximum oxygen uptake (VO2max) has been suggested a stronger risk factor for cardiovascular disease and mortality than high blood pressure, overweight, smoking, and hyperlipidemia[3].

Patients with RA have lower cardiopulmonary fitness, with a reduction of 20% in VO2max compared to age-matched healthy controls[4]. There can be several reasons for the low VO2max in patients with RA, for example, cardiovascular effects of the inflammatory process. However, a probable reason is low physical activity level of patients with RA. The general recommendation for health-enhancing physical activity is: activity/exercise corresponding to a moderate intensity for ≥150 minutes/week or activity/exercise at vigorous intensity for ≥75 minutes/week[5]. Only 36% of RA patients report physical activity at these levels[6]. Indeed, regular physical activity is a common problem, as only a minority of the Swedish population successfully follow recommendations, when objectively assessed using an accelerometer[7].

Atherosclerosis and arterial stiffness are commonly observed in RA [8] and are associated with increased risk of cardiovascular disease. Atherosclerosis in RA is linked to the inflammation driving the rheumatoid disease along with general risk factors, such as low physical activity level, high body mass index (BMI), hypertension and smoking. The gold standard measurement of stiffness in larger arteries is the aortic pulse wave velocity (PWV). Pulse wave velocity increases with age, even in the absence of clinical disease. Studies indicate that aerobic exercise diminishes arterial stiffness[9].

Metabolic and inflammation profiles. RA is characterized by chronic inflammation, with increased production of pro-inflammatory cytokines. Adipokines are metabolic and inflammatory factors that are released in adipose tissue. A well-characterized adipokine is leptin, which is associated with disease activity in RA. Reduction of risk in cardiovascular disease through physical activity has been associated with improved inflammatory and lipid profiles and improved insulin resistance. Changes in these metabolic and inflammatory factors will be examined.

Cardiorespiratory exercise. Regular physical activity and exercise have been shown to confer multiple health effects and to decrease mortality from cardiovascular diseases, stroke, cancer and diabetes. Therefore, the general guidelines related to physical activity for healthy people are currently also recommended for patients with rheumatic diseases [10] and physically active people with risk of clinical coronary artery disease[11].

High-intensity interval aerobic exercise. Physical activity is any bodily movement that increases energy expenditure, while exercise is structured and planned physical activity.

Moderate-intensity aerobic exercise is defined as achieving 60%–74% of the maximum heart rate, while high-intensity level is 75%–94% of the maximum heart rate. High-intensity interval exercise is a relatively new mode of exercise, with alternating short intervals of high and low intensity exercise. The target heart rate during the high-intensity bouts is 90%–95% of the maximum heart rate[12]. This exercise mode has attracted intense interest from the healthy population and among patients with different diseases. One reason for this is that high-intensity exercise is time-efficient and results in a greater improvement in VO2max than moderate continuous exercise[13]. This is important when aiming to prevent cardiovascular disease, since the risks for cardiovascular morbidity and mortality decrease for every increment in 1 ml of VO2max[14].

Exercise interventions for patients with RA are commonly conducted at low or moderate intensities[10], while studies of high-intensity aerobic exercise in RA are scarce. However, a small sample (N=12) with younger (mean age, 33 years) patients with either juvenile RA or RA showed tolerance to high-intensity exercise[15], although no firm conclusions could be drawn.

Our previous studies Our long-term goal is to improve cardiopulmonary function and health for patients with rheumatic disease. The proposed project is based on our previous research on physical activity/ exercise in general [7, 11, 14, 16] and in patients with rheumatic diseases in particular[17-19]. We showed that patients with axial spondyloarthritis (axSpA) can manage aerobic exercise at high intensity[18]. VO2max and disease activity scores [18] as well as fatigue, sleep and mood were improved in the patients with axSpA as a result of the high-intensity exercise[20]. All of these benefits are of major importance to cope with rheumatic disease in daily living. The patients reported increased physical activity at a moderate-to-high intensity level at the 1-year follow-up[19], showing that they motivated for long-term exercise, which is an important outcome. Therefore, we deem it important to study the effects and feasibility of high-intensity interval exercise also for patients with RA.

A pilot study (n=4) was conducted, and it showed positive response of patients with RA engaged in in HIIT program. All patients managed the high intensity exercise level. As patients with RA have an increased risk of cardiovascular disease, and exercise at a high intensity can involve greater risks[11, 16], their risk profile will be taken to account. We will examine the patients using the European Sport Cardiology algorithm, designed for risk evaluation of patients with risk of cardiovascular disease[11]. Pedagogic modes, such as person-centered guidance [17] and motivational support[18, 19], which have previously been shown to contribute to successful outcomes, will be applied in the planned study.

The overarching objective of this study is to evaluate the effects of high-intensity interval aerobic training (HIIT) and strength exercise on cardiovascular health, physical fitness and general health for patients with RA who are receiving the standard of care.

We hypothesize that a 12-week high-intensity interval exercise program will provide substantial improvements in the cardiovascular health, physical fitness and general health of patients with rheumatoid arthritis (RA).

Study design. A randomized controlled intervention trial (0–12 weeks).

Patients
Criteria for inclusion. Patients with established RA diagnosed according to ACR/EULAR 1987/2010 criteria, which comprise clinical examinations of joints, radiological and laboratory measurements. Disease duration >1 year, age range 20–60 years, stable medication on anti-rheumatic drugs for >3 months, and low-to-moderate disease activity (<5.1) according to the Disease activity score 28 (DAS28).

Criteria for exclusion. To cater for the safety of patients participating in this study, it is necessary to exclude patients with other severe diseases that may be associated with adverse events or restrict participation in high-intensity exercise, such as cerebrovascular diseases,

diabetes, severe hypertension, chronic obstructive pulmonary disease, and other severe pulmonary diseases[11, 16]. Screening for exclusion criteria is conducted in several phases of the examination, and include electrocardiogram, CPET, and pulse wave velocity test. Other exclusion criteria are arthroplasty of large joints, inability to manage exercise test (CPET), pregnancy, already participating in regular aerobic or strength exercise at high intensity for >1 hour/ week during the last 6 months, inability to speak or read Swedish.

Procedure

Recruitment. In total, 88 patients (see power calculation) with established RA will be recruited from the rheumatology units at Sahlgrenska University Hospital in Gothenburg and Mölndal, Uddevalla Hospital through the Swedish Rheumatology Quality Register (SRQ).

Oral (by telephone) and written information about the study will be presented to potential participants. Screening will involve assessment of inclusion and exclusion criteria for each subject, according to previously established screening protocol for risk factors of cardiovascular diseases before participation in sports or intense exercise[11, 16]. Those fulfilling criteria will be invited to Clinical Rheumatology Research unit at Sahlgrenska University Hospital. Information about age, gender, disease duration, and pharmacologic treatments will be gathered through structured interviews, medical records and SRQ.

Clinical examinations by a rheumatologist will comprise the general health status and evaluations of rheumatoid disease and comorbidities. Blood samples will be taken, and an electrocardiogram (ECG) will be conducted. A physical assessment with submaximal bicycle test and muscle function will be conducted to determine if the patient can manage the maximal CPET. Patients will be asked to fill in a battery of questionnaires. Thereafter, patients will be referred for the CPET of VO2max and the PWV test for screening for risk of adverse events.

Statistics. To achieve 80% power to detect 10% difference in change between the groups in VO2max, a total of 77 participants needs to be recruited. The calculation of baseline (34 ml/min and SD of difference: 5.0) is based on a previous study (Bilberg, Rheumatol 2005). A total of 88 patients will be recruited to compensate for 20% dropouts. Within- and between-group differences will be analyzed by parametric statistics. If major deviations within the normal range are detected, we will consider data transformation or non-parametric statistics. A statistical advisor will be consulted for all the analyses.

Randomization will be conducted in blocks of four subjects by a computer-generated sequence prepared by a statistician. Concealed, sequentially numbered, sealed, opaque envelopes will be opened by a person not involved in the examinations or treatments, who will also inform regarding the group to which the participant is allocated.

Control group (0-12 weeks)

Participants in the control group meet a physiotherapist for one session to introduce the general health recommendations for physical activity [5] and encourage physical activity on moderate intensive level ≥150 minutes/week. Participants are introduced to a home exercise program for improvement of muscle function and balance during the intervention period [5, 17]. Verbal and written instructions are given, according to previous used model[17].

Intervention (0-12 weeks)

The program includes 12 weeks of aerobic and resistance exercises. Two sessions/week are supervised by an experienced physiotherapist, while a third additional session is non-supervised session of the patient´s own choice. The target of the HIIT intervals is 90%–95% of maximum heart rate (13), based on measured maximum heart rate obtained during CPET at baseline. The high-intensity intervals are alternated with recovery phases at 70% of max heart rate. The exercise protocol comprises 10 minutes of warm-up, followed by 25 minutes of exercise, comprising 4 intervals of 4-minute bouts of high-intensity exercise and 3 intervals of 3-minute bouts of active recovery. The session ends with cooling down for 3–5 minutes.

The exercise is initiated with an individual session, at moderate exercise intensity, progressing to high intensity according to individual capacity. Exercise sessions are modified according to present health at the time of the session (actual symptoms, lack of energy, stress, etc.). Exercise guidance follows principles of self-efficacy and person-centeredness, applying supervision, identification of possible limitations and barriers, exercise diary, a portable heart rate monitor, and continuous dialogue with individual feed-back by a physiotherapist.

Thus, the exercise program is continuously monitored, adapted to each participant and conducted with rigorous guidance, whereby a physiotherapist follows each patient´s heart rate, symptoms, and perceptions. An individual heart rate sensor (Polar H10) connected to a training application loaded in the patient´s cellphone (Polar Beat) and used at each exercise session for monitoring heart rate. Two physiotherapists trained in cardiopulmonary resuscitation (CPR) are present at each session for safety reasons. A mobile intensive care group from the intensive care unit is available less than 5 minutes away from the exercise facilities at the hospitals. Resistance exercise sessions of 20 minutes each include large muscle groups in lower and upper extremities and trunk: 8–10 repetitions with 2–3 sets, with or without equipment.

Primary outcome
Cardiopulmonary function (0 -12 weeks) is assessed by weight adjusted VO2max (ml/kg/min), obtained during progressively increasing work on a bicycle, during which gas exchange will be analyzed, called the cardiopulmonary exercise test (CPET). Registration of ECG data, heart rate and blood pressure will be conducted. A well-established clinical protocol will be applied, and this test is the gold standard when measuring improvement in physical capacity and is directly related to cardiovascular function. The test will be performed in the SU/Clinical Physiology laboratory, as a previous exercise study, for safety reasons and as an outcome measure [17].

Secondary outcomes
Metabolic factors (0-12 week). Lipid status is assessed by changes in blood levels of triglycerides, high-density lipoprotein (HDL), low-density lipoprotein (LDL) and total cholesterol. Blood samples are drawn after eight hours of fasting.
Physical function (0-12 week). Muscle function will be assessed using the 1-minute Sit-to-Stand test and using a dynamometer to measure the grip strength of the hand. Both tests are reliable and have been validated for patients with RA.
Anthropometry measurements (0-12 weeks) will be assessed with body weight and body height, BMI-score (kg/m2) and waist circumference.
Resting blood pressure is recorded with an ambulatory blood pressure monitor with the patient in a seated position, where the lowest value out of two was recorded.
Disease activity (0-12 week) measured using DAS28, a gold standard in rheumatology, based on clinical assessment of 28 joints (swollen, tender), patients´ rating of health (VAS), and sedimentation rate (ESR). A score <5.1 indicates low-to-moderate disease activity and <2.6 indicate remission.
Patient-administered questionnaires on health, symptoms and physical activity are well-established, reliable and valid instruments for patients with RA, and they have been applied in our previous studies.
Pain and global health (0-12 week) are rated on a Visual Analogue Scale (VAS).
Physical activity (0-12 week) is reported on a standardized questionnaire, called the Leisure Time Physical Activity instrument (LTPAI).
Changes in symptoms (12-week) is assessed with the patient global impression of change (PGIC) questionnaires.
Feasibility has previously been evaluated as adherence to the exercise protocol [10]. In this study, will document: all the reasons for declining to participate and for exclusions during the screening process, attendance at exercise sessions; adherence to the study protocol (maximum heart rate, average heart rate); and possible adverse effects.

Ethics
Rigorous control of safety is applied during the screening process as well as when planning and

leading the exercise. Integrity of patients is protected by coding and anonymizing the data. An application to the National Ethics Committee in Sweden will be submitted for approval of the study plan. Data Protection Office at SU will be contacted for GDPR. Incidental findings with regard to cardiovascular health will be remitted to the Cardiology clinic by JB. Other unexpected findings related to the subjects´ health will be addressed by JB who will contact the relevant specialist clinic or general practitioner. JB will contact the Rheumatology clinic of the patient regarding unexpected findings relating to the patient's rheumatic disease.

Research environment and Research facilities relevant to the project
The research team combines three units at the University of Gothenburg (GU), The Sahlgrenska Academy: 1) Institute of Neuroscience and Physiology, Section of Health and Rehabilitation (KM, AB); 2) Institute of Medicine, Department of Molecular and Clinical Medicine (SS, MB); and 3) Institute of Medicine, Department of Rheumatology and Inflammation Research (JB). The clinical units are Rheumatology/SU, Physiotherapy/SU and Rheumatology/Uddevalla.

Coordination. The recruitment will be conducted by Annelie Bilberg and Jan Bjersing together, with tight weekly cooperation. AB will coordinate the examinations and the treatments both for the intervention- and control group. Site visits will be conducted, and telephone meetings will be held each or each other week.

Clinical environment. Patients will be recruited from rheumatology units at Sahlgrenska University Hospital (SU), and from Uddevalla hospitals. Clinical examinations are conducted at the Clinical Research Facility (CRF) in SU/Rheumatology (JB, AB), and at SU/ Clinical physiology laboratory (SS). Exercise interventions are to be conducted at physiotherapy units at SU and Uddevalla hospitals, by using exercise equipment required at these facilities All the physiotherapists will receive appropriate training and education before the start of the project by the project leader (AB).

Personal resources, competence and role in the project
Annelie Bilberg (AB), Senior Physiotherapist, PhD. Working clinically as a physiotherapist at the rheumatology unit, Sahlgrenska University Hospital, Gothenburg and affiliated to Institute of Neuroscience and Physiology/ Physiotherapy. Coordinator for physiotherapists within rheumatology in the Western region of Sweden, which will facilitate coordination of this study, and long-term implementation of the exercise program in clinic. Clinical expert in exercise and rheumatic disease. Project leader and coordinator of the present study, with responsibility for recruiting the patients, as well as the clinical examinations and coordination of all the examinations and treatments for the participants in the study. She will educate and support the physiotherapists acting as leaders at the three study sites in the Western region of Sweden.
Jan Bjersing (JB), MD PhD, Associate Professor and specialist in rheumatology. JB is employed at SU/Rheumatology, including a 20% adjunct lectureship at GU, The Sahlgrenska Academy. Will be responsible for the recruitment and screening of patients. He has experience from several clinical studies involving studies of biomarkers during exercise.
Kaisa Mannerkorpi (KM), PhD and Professor in physiotherapy at the Institute of Neuroscience and Physiology, Section of Health and Rehabilitation, Unit of Physiotherapy. She has supervised seven previous exercise studies. Expert in personal centered health. She is responsible for the personal centered support for long-term exercise after end of the supervised HIIT in the intervention group.
Sara Svedlund (SS), Care unit Chief Physician, MD, PhD, Associate Professor at the Department of Clinical Physiology/SU, and Institute of Medicine, Department of Molecular and Clinical Medicine. Responsible for the execution, interpretation and analysis of the CPET and PWV data in this study. Runs an academic core laboratory that uses different modalities for cardiovascular characterization.
Mats Börjesson (MB), Professor of Sports Physiology and MD, Specialist in Cardiology and Internal medicine. Special research interest in health benefits and potential risks of physical activity and sports (Sports Cardiology). Head of Center for Health and Performance Laboratory at University of Gothenburg, Sweden. He is responsible for the research design

from a cardiological perspective including safety aspects and participates in the analysis of the data.

References

1. Avina-Zubieta JA, Thomas J, Sadatsafavi M, Lehman AJ, Lacaille D: Risk of incident cardiovascular events in patients with rheumatoid arthritis: a meta-analysis of observational

studies. *Annals of the rheumatic diseases* 2012, 71(9):1524-1529.
2. Nicola PJ, Crowson CS, Maradit-Kremers H, Ballman KV, Roger VL, Jacobsen SJ, Gabriel SE: Contribution of congestive heart failure and ischemic heart disease to excess mortality in rheumatoid arthritis. *Arthritis Rheum* 2006, 54(1):60-67.
3. Ross R, Blair SN, Arena R, Church TS, Despres JP, Franklin BA, Haskell WL, Kaminsky LA, Levine BD, Lavie CJ *et al*: Importance of Assessing Cardiorespiratory Fitness in Clinical Practice: A Case for Fitness as a Clinical Vital Sign: A Scientific Statement From the American Heart Association. *Circulation* 2016, 134(24):e653-e699.
4. Liff MH, Hoff M, Fremo T, Wisloff U, Thomas R, Videm V: Cardiorespiratory fitness in patients with rheumatoid arthritis is associated with the patient global assessment but not with objective measurements of disease activity. *RMD open* 2019, 5(1):e000912.
5. Piercy KL, Troiano RP: Physical Activity Guidelines for Americans From the US Department of Health and Human Services. *Circ Cardiovasc Qual Outcomes* 2018, 11(11):e005263.
6. Arne M, Janson C, Janson S, Boman G, Lindqvist U, Berne C, Emtner M: Physical activity and quality of life in subjects with chronic disease: chronic obstructive pulmonary disease compared with rheumatoid arthritis and diabetes mellitus. *Scand J Prim Health Care* 2009, 27(3):141-147.
7. Ekblom-Bak E, Olsson G, Ekblom O, Ekblom B, Bergstrom G, Borjesson M: The Daily Movement Pattern and Fulfilment of Physical Activity Recommendations in Swedish Middle-Aged Adults: The SCAPIS Pilot Study. *PloS one* 2015, 10(5):e0126336.
8. Ambrosino P, Lupoli R, Di Minno A, Tasso M, Peluso R, Di Minno MN: Subclinical atherosclerosis in patients with rheumatoid arthritis. A meta-analysis of literature studies. *Thromb Haemost* 2015, 113(5):916-930.
9. Yoshizawa M, Maeda S, Miyaki A, Misono M, Saito Y, Tanabe K, Kuno S, Ajisaka R: Effect of 12 weeks of moderate-intensity resistance training on arterial stiffness: a randomised controlled trial in women aged 32-59 years. *British journal of sports medicine* 2009, 43(8):615-618.
10. Rausch Osthoff AK, Niedermann K, Braun J, Adams J, Brodin N, Dagfinrud H, Duruoz T, Esbensen BA, Gunther KP, Hurkmans E *et al*: 2018 EULAR recommendations for physical activity in people with inflammatory arthritis and osteoarthritis. *Annals of the rheumatic diseases* 2018, 77(9):1251-1260.
11. Borjesson M, Dellborg M, Niebauer J, LaGerche A, Schmied C, Solberg EE, Halle M, Adami E, Biffi A, Carre F *et al*: Recommendations for participation in leisure time or competitive sports in athletes-patients with coronary artery disease: a position statement from the Sports Cardiology Section of the European Association of Preventive Cardiology (EAPC). *Eur Heart J* 2019, 40(1):13-18.
12. Karlsen T, Aamot IL, Haykowsky M, Rognmo O: High Intensity Interval Training for Maximizing Health Outcomes. *Prog Cardiovasc Dis* 2017, 60(1):67-77.
13. Helgerud J, Hoydal K, Wang E, Karlsen T, Berg P, Bjerkaas M, Simonsen T, Helgesen C, Hjorth N, Bach R *et al*: Aerobic high-intensity intervals improve VO2max more than moderate training. *Medicine and science in sports and exercise* 2007, 39(4):665-671.
14. Ekblom-Bak E, Ekblom B, Soderling J, Borjesson M, Blom V, Kallings LV, Hemmingsson E, Andersson G, Wallin P, Ekblom O: Sex- and age-specific associations between cardiorespiratory fitness, CVD morbidity and all-cause mortality in 266.109 adults. *Prev Med* 2019, 127:105799.
15. Sandstad J, Stensvold D, Hoff M, Nes BM, Arbo I, Bye A: The effects of high intensity interval training in women with rheumatic disease: a pilot study. *European journal of applied physiology* 2015, 115(10):2081-2089.
16. Borjesson M, Urhausen A, Kouidi E, Dugmore D, Sharma S, Halle M, Heidbuchel H, Bjornstad HH, Gielen S, Mezzani A *et al*: Cardiovascular evaluation of middle-aged/ senior individuals engaged in leisure-time sport activities: position stand from the sections of exercise physiology and sports cardiology of the European Association of Cardiovascular Prevention and Rehabilitation. *Eur J Cardiovasc Prev Rehabil* 2011, 18(3):446-458.
17. Lange E, Kucharski D, Svedlund S, Svensson K, Bertholds G, Gjertsson I, Mannerkorpi K: Effects of Aerobic and Resistance Exercise in Older Adults With Rheumatoid Arthritis: A Randomized Controlled Trial. *Arthritis care & research* 2019, 71(1):61-70.
18. Sveaas SH, Bilberg A, Berg IJ, Provan SA, Rollefstad S, Semb AG, Hagen KB, Johansen MW, Pedersen E, Dagfinrud H: High intensity exercise for 3 months reduces disease activity in axial spondyloarthritis (axSpA): a multicentre randomised trial of 100 patients. *British journal of sports medicine* 2020, 54(5):292-297.
19. Sveaas SH, Dagfinrud H, Johansen MW, Pedersen E, Wold OM, Bilberg A: Longterm Effect on Leisure Time Physical Activity Level in Individuals with Axial Spondyloarthritis: Secondary Analysis of a Randomized Controlled Trial. *J Rheumatol* 2020, 47(8):1189-1197.
20. Sveaas SH, Dagfinrud H, Berg IJ, Provan SA, Johansen MW, Pedersen E, Bilberg A: High-Intensity Exercise Improves Fatigue, Sleep, and Mood in Patients With Axial Spondyloarthritis:

Secondary Analysis of a Randomized Controlled Trial. *Phys Ther* 2020, 100(8):1323-1332.
