# Supplementary material for: High-intensity interval training improves cardiovascular and physical health in patients with rheumatoid arthritis: a multicentre randomised controlled trial
Source: Br J Sports Med. 2024 Aug 23;58(23):e108369. doi: 10.1136/bjsports-2024-108369 (PMC11672065; doi:10.1136/bjsports-2024-108369)
Supplement: online supplemental file 2 [file bjsports-58-23-s002.pdf]

Table 4. Exploratory interaction analysis on VO_2max for the total study population (N=87) and baseline variables

	Interaction *p*-value	Mean diff of change (95%CI)	*p*-value
Age	0.3948		
Gender	0.0005		
Female		2.36 (1.13; 3.59)	0.0003
Male		9.67 (4.25; 15.09)	0.0003
Weight, kg	0.048		
≤70		2.72 (0.82; 4.63)	0.0069
>70		4.35 (1.91; 6.80)	0.0008
Length, cm	0.0002		
≤170		1.72 (-0.01; 3.45)	0.0510
>170		5.74 (3.44; 8.04)	<.0001

Interaction analyses between the total study population and baseline variables regarding primary efficacy variable as dependent
