# Supplementary material for: High-intensity interval training improves cardiovascular and physical health in patients with rheumatoid arthritis: a multicentre randomised controlled trial
Source: Br J Sports Med. 2024 Aug 23;58(23):e108369. doi: 10.1136/bjsports-2024-108369 (PMC11672065; doi:10.1136/bjsports-2024-108369)
Supplement: online supplemental file 3 [file bjsports-58-23-s003.pdf]

Table 5. The per procol analyses for the changes in secondary outcomes from baseline to 3 months follow-up between the groups

	Intervention group (n=33)		Control group (n=44)		Between group	
	BL<br>Means (SD)	3 Month<br>Means (SD)	BL<br>Means (SD)	3 Month<br>Means (SD)	Mean diff of change<br>BL to 3M<br>(95% CI)<br>*p*-value	Effect size
VO2 mL/min	2108 (450.0)	2378 (581.0)	2013 (569.0)	1988 (579.0)	292.1 (195.6; 389.9)<br><0.0001	1.45
O2-pulse mL/beat/min	12.2 (2.6)	13.9 (3.5)	11.9 (3.1)	12.1 (3.4)	1.61 (1.05; 2.17)<br><0.0001	1.39
VEmax L/min	87.6 (19.3)	93.5 (22.3)	82.1 (22.2)	81.2 (24.9)	7.21 (1.2; 13.21)<br>0.016	0.57
RER	1.18 (0.08)	1.15 (0.09)	1.20 (0.09)	1.20 (0.10)	-0.016 (-0.04; 0.01)<br>0.018	0.59
HRmax, beats/min	173.9 (11.1)	171.9 (11.9)	169.4 (16.5)	164.8 (16.3)	0.87 (-2.82; 4.45)<br>0.65	0.12
Systolic BP	124.0 (17.6)	121.2 (16.4)	123.9 (15.9)	124.4 (17.7)	-2.39 (-8.23; 3.47)<br>0.43	0.19
Diastolic BP	74.9 (11.6)	73.5 (10.3)	74.5 (10.5)	75.4 (11.3)	-1.72 (-5.03; 1.58)<br>0.31	0.25
Grip strength, N	228.2 (79.4)	262.4 (90.7)	213.1 (87.4)	216.8 (93.0)	32.2 (5.2; 58.9)<br>0.02	0.56
One-minute STS, no	25.0 (5.4)	30.7 (5.1)	25.0 (6.4)	25.9 (6.7)	4.28 (2.60; 5.94)<br><0.0001	1.20
Anthropometry						
Weight, kg	80.0 (16.5)	79.5 (15.5)	78.5 (19.1)	78.2 (19.2)	-0.55 (-1.47; 0.39)<br>0.24	0.28
BMI, kg/m2	27.2 (5.8)	27.0 (5.5)	27.1 (5.3)	26.9 (5.1)	-0.17 (-0.48; 0.15)<br>0.29	0.26
Waist circumference, cm	90.9 (14.7)	87.1 (12.2)	89.1 (15.2)	88.7 (14.4)	-3.56 (-6.16; -0.94)<br>0.0098	0.64

Serum lipids						
S-TC	5.34 (1.43)	5.19 (1.41)	5.16 (1.00)	5.22 (0.95)	-0.15 (-0.39; 0.08)<br>0.2	0.31
S-HDL	1.62 (0.41)	1.61 (0.36)	1.58 (0.39)	1.59 (0.39)	-0.02 (-0.11; 0.08)<br>0.71	0.09
S-LDLmm/L	3.64 (1.26)	3.45 (1.26)	3.45 (0.89)	3.47 (0.78)	-0.11 (-0.31; 0.1)<br>0.29	0.26
S-TG mm/L	0.87 (0.39)	0.94 (0.38)	0.99 (0.42)	0.95 (0.38)	0.05 (-0.07; 0.18)<br>0.39	0.21
Disease activity						
DAS-28	2.0 (0.90)	1.9 (0.84)	2.0 (1.18)	2.3 (1.33)	-0.27 (-0.7; 0.1)<br>0.15	0.35
ESR	10.8 (12.0)	11.8 (10.9)	11.7 (10.1)	13.5 (11.4)	-0.76 (-3.57; 2.1)<br>0.62	0.13
CRP	2.2 (3.27)	2.5 (3.84)	2.3 (3.07)	2.8 (3.33)	-0.15 (-1.66; 1.33)<br>0.87	0.05
VAS-Global, 0-100	21.1 (20.0)	16.6 (16.7)	18.5 (19.0)	29.5 (26.9)	-16.20 (-26.30; -6.40)<br>0.0015	0.77
VAS-Pain, 0-100	19.9 (18.6)	17.4 (16.8)	20.1 (20.1)	21.6 (22.4)	-5.16 (-15.0; 4.48)<br>0.30	0.25

Values are shown as mean and SD unless indicating otherwise. VO2mL/min, maximal oxygen uptake; O2-puls, oxygen pulse, VEmax, ventilatory maximal; RER; respiratory exchange ratio; HRmax, maximal heart rate; BP, blood pressure at rest; One-minute STS, One-minute Sit-To-Stand test; BMI, body mass index; WCF, waist circumference; Serum levels of S-TG, total cholesterol; S-HDL, high-density lipoprotein; S-LDL, low-density lipoprotein,; S-TC, triglycerides; DAS28, Disease Activity Score in 28 joints; ESR, erythrocyte sedimentation rate; CRP, C reactive protein. Missing values at month 3 in the CG, CRF (n=8), BP (n=6), grip strength (n=6), STS (n=6), Serum lipids (n=6), Anthropometry (n=6), DAS28 (n=7), ESR and CRP (n=6).
