# Supplementary material for: High-intensity interval training improves cardiovascular and physical health in patients with rheumatoid arthritis: a multicentre randomised controlled trial
Source: Br J Sports Med. 2024 Aug 23;58(23):e108369. doi: 10.1136/bjsports-2024-108369 (PMC11672065; doi:10.1136/bjsports-2024-108369)
Supplement: online supplemental file 4 [file bjsports-58-23-s004.pdf]

Table 6. Changes in secondary key outcomes from baseline to 3 months follow-up between the groups for the women and men separately

	Intervention group BL Means (SD)	Intervention group 3 Month Means (SD)	Control group BL Means (SD)	Control group 3 Month Means (SD)	Between group Mean diff of change BL to 3 Month (95% CI) *p*-value	Effect size
**Women**	(n=37)	(n=36)	(n=36)	(n=30)		
VO_2max, mL/kg/min	25.7 (5.4)	28.1 (6.3)	25.0 (5.7)	24.3 (5.4) (n=27)	2.37 (1.14; 3.60) 0.0002	1.00
Grip strength, N	202.7 (60.4)	227.8 (67.7)	197.2 (62.4)	185.1 (58.1)	35.30 (15.20; 55.50) 0.0006	0.85
One-minute STS, no	23.5 (5.1)	30.1 (5.2)	24.8 (6.3)	25.3 (6.3)	5.27 (3.47; 7.06) <0.001	1.45
DAS28	2.2 (0.83)	2.1 (0.70)	2.1 (1.25)	2.5 (1.36)	-0.36 (-0.77; 0.04) 0.08	0.44
**Men**	(n=6)	(n=5)	(n=8)	(n=8)		
VO_2max, mL/kg/min	29.3 (4.0)	36.5 (5.0)	33.1 (6.2)	30.3 (5.5)	9.48 (5.65; 17.06) 0.0016	1.57
Grip strength, N	287.8 (132.3)	408.2 (67.6)	284.5 (142.7)	335.9 (105.9)	25.2 (-89.0; 118.3) 0.65	0.26
One-minute STS, no	24.5 (7.9)	32.2 (5.8)	25.9 (7.3)	28.3 (8.0)	4.23 (-2.50; 11.00) 0.18	0.82
DAS28	1.0 (0.76)	0.9 (0.92)	1.6 (0.61)	1.4 (0.75)	0.17 (-0.40; 0.76) 0.51	0.36

Values are shown as mean and SD unless indicating otherwise. VO_2max, weight corrected maximal oxygen uptake; One-minute STS, One-minute Sit-To-Stand test; DAS28, Disease Activity Score in 28 joints
